# Regulation of CD8^+^ T Cells and Antitumor Immunity by Notch Signaling

**DOI:** 10.3389/fimmu.2018.00101

**Published:** 2018-01-30

**Authors:** Shin-ichi Tsukumo, Koji Yasutomo

**Affiliations:** ^1^Department of Immunology and Parasitology, Graduate School of Medicine, Tokushima University, Tokushima, Japan

**Keywords:** Notch, T cells differentiation, tumor immunity, CD8^+^ T cells, granzyme B

## Abstract

Cancer immunosurveillance is critical for the elimination of neoplastic cells. In addition, recent advances in immunological checkpoint blockade drugs have revealed the importance of the immune system in cancer treatment. As a component of the immune system, CD8^+^ T cells have important roles in suppressing tumors. CD8^+^ T cells can kill tumor cells with cytotoxic molecules, such as granzymes and perforin. IFNγ, which is produced by CD8^+^ T cells, can increase the expression of MHC class I antigens by tumor cells, thereby rendering them better targets for CD8^+^ T cells. IFNγ also has crucial functions in enhancing the antitumor abilities of other immune cells. Therefore, it has been hypothesized that antitumor immunity could be improved by modulating the activity of CD8^+^ T cells. The Notch pathway regulates CD8^+^ T cells in multiple ways. It directly upregulates mRNA expression of granzyme B and perforin, enhances differentiation toward short-lived effector cells, and maintains memory T cells. Intriguingly, CD8^+^ T cell-specific Notch2 deletion impairs antitumor immunity, whereas the stimulation of the Notch pathway can increase tumor suppression. In this review, we will summarize the roles of the Notch pathway in CD8^+^ T cells and discuss issues and implications for its use in antitumor immunity.

## Introduction

To suppress tumor cell growth, animals use their cell-intrinsic antitumor system, which is regulated by tumor suppressor genes. A second line of defense against tumors includes the immune system itself (1, 2). Acquired immune cells, especially CD8^+^ T cells, can detect and kill tumors through the latter’s expression of abnormal antigens derived from mutated, overexpressed or ectopically expressed molecules. Innate immune cells also have important roles in the antitumor system. For example, NK cells can target tumors by recognizing the expression of MHC class Ib proteins induced by cellular transformation or the lack of MHC class I molecules. Many efforts have been devoted to treating cancer by enhancing immunosurveillance.

Many efforts have been made to enhance antitumor immunity. For example, administration of cytokines, such as type I interferon, IL-2, and IL12, or TLR agonists such as BCG and imiquimod is employed to non-specifically stimulate immune system (3). Vaccine against tumors is also examined to treat them; irradiated tumor cells or selected antigens specifically expressed in tumors are used to increase tumor-specific T cell response (4). In addition, *in vitro* activated and expanded T cells, which can recognize tumors, are adoptively transferred to patients to increase tumor-specific immunity (5). Notably, recent advances in the development of checkpoint blockade drugs, such as antibodies to PD-1 and CTLA-4, indicate that this field of research is indeed promising (6, 7). To further improve immunotherapy, we need a better understanding of the antitumor immune system.

The Notch pathway is an evolutionarily conserved signaling pathway that regulates various biological systems, including a wide variety of functions of peripheral T cells (8–10). In mammals, the Notch system consists of four receptors (Notch1 to 4) and five ligands (Dll1, 3, 4, and Jagged1, 2). When the receptor is stimulated by the ligand, it is cleaved by an ADAM-family metalloprotease and subsequently the γ-secretase complex, and its cytoplasmic domain is translocated into the nucleus. The cytoplasmic domain then binds to DNA binding protein RBPJκ (encoded by *Rbpj*) and co-activator MAML, leading to transcriptional regulation of specific target genes.

**Figure 1:**
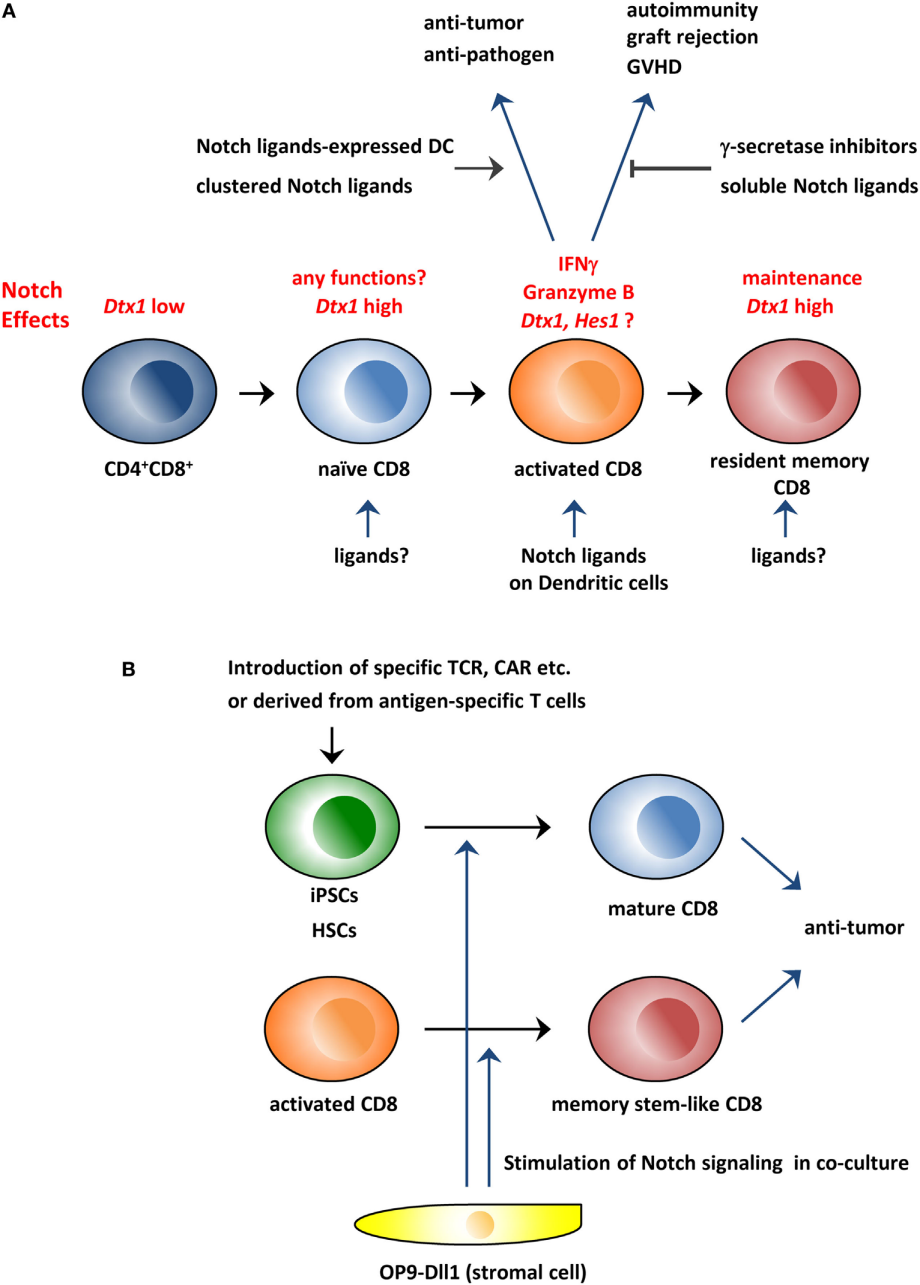
Schematic overview of the roles of the Notch pathway in CD8^+^ T cells and its application to immunotherapy. **(A)** The Notch pathway is stimulated during CD8^+^ T cell activation and is required for the production of effector molecules, such as IFNγ and granzyme B. Therefore, the modulation of the Notch pathway could be used to treat various diseases in which CD8^+^ T cells are involved. In addition, studies indicate that the Notch pathway is active in resting naïve and memory T cells in which the pathway is reportedly needed for the maintenance of these cells. **(B)** Coculture with Dll1-expressing OP9 stromal cells can generate CD8^+^ T cells from hematopoietic stem cells or iPSCs *in vitro*. In addition, the coculture system can generate memory stem cell-like T cells from activated CD8^+^ T cells. These *in vitro* generated CD8^+^ T cells could be superior reagents for antitumor immunity. GVHD, graft-versus-host disease; CAR, chimeric antigen receptor; iPSCs, induced pluripotent stem cells; HSCs, hematopoietic stem cells.

Research into the physiological roles of the Notch pathway in peripheral T cells has mainly focused on CD4^+^ T cells. The Notch pathway regulates CD4^+^ T cell differentiation, cytokine production, proliferation, and/or survival, although some of the data among the papers are in disagreement (8, 9). For example, Tanigaki et al. reported that *Rbpj*-deficient CD4 T cells showed decreased Th2 and increased Th1 in *in vivo* and *in vitro* experiments (11). Similarly, Amsen et al. reported Th2 differentiation was dependent on the Notch pathway by using *Notch1/2*-double deficient mice in addition to *Rbpj* (12). On the other hand, Auderset et al. reported that *Notch1* and *2* were required for Th1 differentiation in anti-*Leishmania major* immunity, while *Rbpj*-deficiency did not show any significant effects (13). The causes of these apparent differences have not been resolved. It is possible that the functions of Notch pathway are highly context-dependent in T cells. In this review, we will summarize research into the physiological roles of the Notch pathway in CD8^+^ T cells and discuss its potentials for antitumor immunity (Figure 1).

## The Physiological Roles of the Notch Pathway in CD8^+^ T Cells

To elucidate the roles of Notch in CD8^+^ T cells, studies have analyzed mice in which the Notch pathway has been knocked out. Maekawa et al. reported that CD8^+^ T cell-specific (E8I-cre) *Notch2* deletion led to decreased expression of *Gzmb* (encoding granzyme B) and increased sensitivity to *Trypanosoma cruzi* infection (14). This mouse also showed a significant loss of CTL activity against antigen-pulsed cells *in vivo*. They further showed that Notch2 and RBPJκ directly bound to *Gzmb* and *Prf1* (encoding perforin) promoters in combination with the transcription factor CREB and activated their transcription.

Backer et al. described an influenza virus infection model in which T cell-specific (CD4-cre) *Notch1/2*-double KO mice showed almost complete loss of short-lived effector CD8^+^ T cells (SLECs) that possess the KLRG1^+^CD127^−^ phenotype. On the other hand, the overall ratio of antigen-specific CD8^+^ T cells to that of KLRG1^−^CD127^+^ memory precursor effector cells (MPECs) was moderately increased (15). They also confirmed this phenotype was present in *Rbpj* KO mice. Then, they analyzed the transcriptome of activated CD8^+^ T cells, and showed that more than 40% of SLEC-specific genes were decreased in *Notch1/2* KO cells, indicating that the Notch pathway was a critical regulator of SLEC differentiation. In addition, they also found that the Notch pathway was required for the upregulation of CD25 (IL-2Rα chain) and T-bet proteins, both of which are critical regulators of SLEC differentiation. Furthermore, they showed that T-bet overexpression enhanced SLEC differentiation in *Notch1/2* KO CD8^+^ T cells, while the active form of Notch1 could not do so in *Tbx21* (encoding T-bet) KO cells, suggesting that T-bet is a critical regulator downstream in the Notch pathway.

Similar results were reported by another laboratory. Mathieu et al. used CD8 T cell-specific *Notch1/2* KO mice and showed a reduction of the ratio of SLECs after *Listeria monocytogenes* infection (16). However, they found that the absolute cell number of SLECs was not reduced, and the reduction of the ratio was instead due to an increased number of MPECs and early effector cells (EECs; KLRG1^−^CD127^−^ cells). On the other hand, when they immunized mice with peptide-pulsed dendritic cells (DCs), they found a severe reduction of SLEC cell number, while MPEC cell numbers were not affected. The reason for this difference was not clear, but it might indicate that the roles of the Notch pathway in CD8^+^ T cells are context-dependent as seen in CD4^+^ T cells (8). As reported in the paper by Backer et al. above, they found that CD25 protein expression was diminished in *Notch1/2* KO cells. However, the expression of T-bet was not affected. Instead, they found that Eomes, which is a paralog of T-bet, was moderately decreased in *Notch1/2* KO cells. Eomes is reportedly required for MPEC differentiation but not for SLEC (17). Thus, the importance of the Eomes reduction in *Notch1/2* KO cells for SLEC differentiation remains to be investigated.

Instead of KO mice, Maillard and colleagues used the dominant negative form of MAML (DN-MAML)-expressing mice and analyzed its effects on CD4^+^ and CD8^+^ T cells in a graft-versus-host disease (GVHD) model (18, 19). They reported that DN-MAML profoundly suppressed GVHD, with reduced production of IFNγ in CD4^+^ and CD8^+^ T cells. In contrast to KO mouse experiments, DN-MAML-expressing CD8^+^ T cells preserved their T-bet and Eomes protein expression. In addition, those cells showed a defect in the activation of Ras/MAPK and NF-κB pathways. Those cells also expressed higher amounts of negative regulators of T cell activation, such as *Dgka, Cblb*, and *Pdcd1*, suggesting that these factors might suppress GVHD.

In addition to the genetic approaches described above, γ-secretase inhibitors, blocking antibodies and soluble Notch ligands have been used to investigate the roles of the Notch pathway in CD8^+^ T cells (20–26). The consensus of these experiments is that the Notch pathway is required for IFNγ production during CD8^+^ T cell activation. On the other hand, the effect on the cell number after the activation of CD8^+^ T cells was controversial. Several papers indicated that γ-secretase inhibitors or soluble Notch ligand (Dll4) suppressed proliferation of CD8^+^ T cells, while their viability was not affected (22–24). Other papers showed that the inhibitors or membrane-bound Notch ligands (Jagged1) did not affect the CD8 T cell number or proliferation after activation (25, 27, 28). In addition, Notch1/2-double KO mice showed that the CD8^+^ T cell number was not affected or even increased when activated *in vivo*, although their differentiation was altered (15, 16). What caused these differences remains elusive. Further examination of the experiment-conditions and the methods of the Notch inhibition should be required in future researches.

Other studies showed that the cell surface expression of Notch1 and 2 was upregulated soon after T cell activation (14, 15, 22, 23, 29, 30). In addition, expression of its ligands (Dll1, Dll4, and/or Jagged1) was also upregulated in activated DCs (15, 21–23, 31). Based on these observations, many researchers have concluded that the Notch pathway is activated early in the process of T cell activation by the ligands on DCs. In fact, it was reported that *Hes1* and/or *Dtx1* (encoding Deltex1), which are well-known targets of the Notch pathway, were upregulated after T cell activation (23, 32). Other papers reported that TCR stimulation caused the cleavage of Notch receptors, indicating that the Notch pathway was activated after T cell activation (20, 33). However, transcriptome analyses clearly show that *Dtx1* is upregulated during the differentiation of CD4^+^CD8^+^ thymocytes to peripheral naïve CD4^+^ and CD8^+^ T cells (Immunological genome project^1^; RCAI RefDIC^2^). We confirmed that this upregulation was dependent on *Notch1/2* and *Rbpj* (unpublished data). Unexpectedly, *Dtx1* is moderately downregulated after TCR activation, according to transcriptome data. Subsequently, its expression returns to a high level during the differentiation to memory cells. On the other hand, *Hes1* expression remains low during activation of naïve and activated cells. These results suggest that the Notch pathway is active in resting T cells. The reason why *Hes1* and *Dtx1* were not upregulated during T cell activation remains unclear. The Notch pathway might not be activated under the conditions of T cell activation used in these studies. Alternatively, the epigenetic status of these gene loci or unknown inhibitor(s) might affect their expression during T cell activation.

Interestingly, recent papers support the hypothesis that the Notch pathway is operational in resting CD4^+^ and CD8^+^ T cells. Maekawa et al. reported that *Rbpj*-deficient CD4^+^ T cells normally expanded after antigen stimulation, but could not survive during the contraction phase. They also found that the injection of γ-secretase inhibitor to mice decreased the number of resting memory T cells (34). Hombrink et al. also reported that *Notch1/2*-deficiency or the treatment with γ-secretase inhibitor decreased CD103^+^ lung-resident memory CD8^+^ T cells in mice (35). These results suggest that the Notch pathway has important roles not only in activating T cells but also in resting cells.

Although some data disagree, an increasing number of reports have demonstrated that the Notch pathway was required for CD8^+^ T cell activation and homeostasis. When and how the Notch pathway works remains to be further investigated, but it is very probable that the manipulation of this pathway could be useful in the treatment of diseases in which the immune system is involved.

## The Notch Pathway in Antitumor Immune Responses

CD8^+^ T cells have important roles in antitumor immunity (1, 7), some of which are dependent upon the Notch pathway. Sugimoto et al. reported that CD8-specific deletion of *Notch2*, but not *Notch1*, led to increased tumor size and decreased survival after tumor-inoculation into mice (36). Zhao et al. reported that ovarian cancer imposed glucose restriction on T cells, leading to high expression of microRNAs *miR-101* and *miR26a*, leading to constrained expression *Ezh2*. *Ezh2* is a suppressor of Notch pathway inhibitors *Numb* and *Fbxw7*. As a consequence, the cancer-induced glucose restriction led to the suppression of the Notch pathway. They also showed that downregulation of *Ezh2* elicited poor antitumor immunity, implying that the Notch pathway was important for antitumor immunity (37). Dai et al. found that *1810011o10Rik* (*Tcim*) was upregulated in intratumoral activated CD8^+^ T cells. They also showed that overexpression of *Tcim* blocked nuclear translocation of the intracellular domain of Notch2 and inhibited the cytotoxic efficacy of CD8^+^ T cells on hepatocellular carcinoma (38). All of these papers confirm that the Notch pathway in CD8^+^ T cells has a critical role in antitumor immunity.

Considering these reports, the manipulation of the Notch pathway in T cells could be a good approach to suppress tumors. Several papers pursued the idea in mouse models. Sugimoto et al. showed that injection of agonistic antibody to Notch2 or Dll1-overexpression in DC augmented antitumor immunity (36). Sierra et al. used intracellular Notch1-expressing mice driven by a granzyme B promoter-cre and flox system. They found that such activation of the Notch pathway in CD8^+^ T cells increased the cytotoxic effects and antitumor response with higher production of IFNγ and granzyme B (39). Thounaojam et al. showed that treatment with the proteasome inhibitor bortezomib caused higher expression of IFNγ in CD8^+^ T cells in tumor-bearing mice, probably through the upregulation of Notch receptors (40). Biktasova et al. reported that administration of clustered Dll1 enhanced IFNγ-producing CD8^+^ T cells and suppressed tumor growth (41). These reports reveal that Notch-targeted immune modulation could be promising. However, Notch receptors are broadly expressed in various types of cells, and the modulation of Notch might be highly context-dependent. In addition, Notch receptors are known as proto-oncogenes themselves (42). Therefore, it is possible that the activation of the pathway could exacerbate some types of tumors. Detailed investigations will be needed to examine the possibility of antitumor treatment targeting this pathway.

The therapy by immune checkpoint blockade is recent advance in antitumor immunotherapy (43). The blocking antibodies to PD-1/PD-L1 and CTLA-4 are broadly used to treat melanoma and other types of tumors. Mathieu et al. reported that Notch directly bound to the promoter region of *Pdcd1* (encoding PD-1) gene and upregulated its mRNA expression in activated CD8^+^ T cells (23). In addition, Yu et al. indicated that γ-secretase inhibitor activated tumor-infiltrating CD8^+^ T cell probably through the downregulation of PD-1 expression (44). These results indicated that the Notch pathway might also have negative effect during CD8^+^ T cell activation. Therefore, it is expected that the antitumor therapy by Notch activation would be more efficient in combination with the blocking antibodies to PD-1 and other inhibitory receptors.

## Generating Antitumor CD8^+^ T Cells *In Vitro* Using the Notch Pathway

In addition to efforts to modulate the Notch pathway *in vivo* to enhance antitumor immunity, there have been *in vitro* attempts to create cytotoxic T cells against tumors. CD8^+^ memory stem cells are reported to have naïve markers, but have self-renewal capacity and can rapidly respond to antigens (45, 46). In addition, they have antitumor capacities exceeding those of central and effector memory T cells (47). Kondo et al. reported that activated CD4^+^ or CD8^+^ T cells could be converted to memory stem cell-like cells when cocultured with Dll1-expressing OP9 stromal cells (OP9-Dll1) (48). They also showed that the resultant memory stem cell-like CD4^+^ and CD8^+^ T cells had superior antitumor abilities relative to naïve, activated or memory T cells when injected into mice.

In addition to peripheral T cells, the Notch pathway is well known for its role in defining the fate of T cells in early stages of differentiation. By coculturing with Dll1-expressing cells, some types of stem cells can be differentiated to T cells *in vitro* (49). There have been several attempts to create large number of tumor-specific CD8^+^ T cells through use of this *in vitro* system. Zhao et al. introduced a tumor antigen-specific TCR into human umbilical cord blood-derived hematopoietic cells and generated T cells by coculture with OP9-Dll1 (50). They showed that those T cells could recognize and kill antigen-pulsed antigen-presenting cells. Vizcardo et al. generated induced pluripotent stem cells (iPSCs) from melanoma antigen-specific human cytotoxic T cells and cultured them on OP9-Dll1 cells. They subsequently stimulated the differentiated CD4^+^CD8^+^ T cells with anti-CD3 antibody to create CD8^+^ single positive T cells (51). They found that those CD8^+^ T cells could respond to the specific melanoma antigen, and had antitumor ability. Themeli et al. introduced a chimeric antigen receptor into iPSCs and generated human T cells targeted against CD19 by using OP9-Dll1 (52). Although the generated T cells showed an innate T cell-like phenotype, those cells had potent antitumor capability specific for CD19-expressing lymphoma cells.

## Conclusion and Future Directions

Emerging evidence indicates that the Notch pathway has important physiological roles in CD8^+^ T cell functions, especially in the production of effector molecules. In addition, recent research points out that the Notch pathway probably works in resting T cells to promote homeostasis. On the other hand, the presence of apparently conflicting data suggests that the roles of the Notch pathway might be highly stage and context dependent. Therefore, it is critical to clarify what determines the functions of the Notch pathway under each condition. Comprehensive analyses of Notch signaling by transcriptomic, proteomic, and ChIP-seq analyses would be helpful to elucidate the differences under each condition.

Given the physiological importance of the Notch pathway, it could prove useful in the optimization of antitumor immunotherapy. However, the manipulation of the pathway should be carefully examined because the roles of the pathway could be context-dependent even in peripheral T cells. Furthermore, Notch receptors and ligands are broadly expressed in many tissues, and the manipulation of the pathway could cause unpredicted outcomes.

As well as the administration of cytokines, TLR agonists and immune checkpoint inhibitors, the activation of the Notch pathway induces non-specific activation of immune system, which could lead to autoimmunity or unwanted inflammation. Tumor-specific activation of immune response has been tried by using vaccination against tumor antigens or adoptive transfer of tumor-specific T cells generated or expanded *in vitro*. As described in this minireview, the Notch pathway is an excellent tool to create large amount of CD8^+^ T cells from iPSCs derived from tumor-specific T cells *in vitro*. In addition, the Notch pathway also can induce memory stem cell-like cells from peripheral T cells. Tuning the culture conditions as well as genetic modification of the cells could be used to create various types of CD8^+^ T cells for cancer immunotherapy. The best combination of non-specific and specific activation of immune responses should be carefully investigated to fight against tumors in various conditions.

## Author Contributions

All authors listed have made a substantial, direct, and intellectual contribution to the work and approved it for publication.

## Conflict of Interest Statement

The authors declare that the research was conducted in the absence of any commercial or financial relationships that could be construed as a potential conflict of interest.

## Footnotes

^1^http://www.immgen.org/index_content.html.

^2^http://refdic.rcai.riken.jp/welcome.cgi.
